# Betulinic Acid Protects From Bone Loss in Ovariectomized Mice and Suppresses RANKL-Associated Osteoclastogenesis by Inhibiting the MAPK and NFATc1 Pathways

**DOI:** 10.3389/fphar.2020.01025

**Published:** 2020-07-07

**Authors:** Jiyong Wei, Yicheng Li, Qian Liu, Yanni Lan, Chengming Wei, Kun Tian, Liwei Wu, Chunbo Lin, Jiake Xu, Jinmin Zhao, Yuan Yang

**Affiliations:** ^1^ Research Centre for Regenerative Medicine, Guangxi Key Laboratory of Regenerative Medicine, Guangxi Medical University, Nanning, China; ^2^ Guangxi Collaborative Innovation Center for Biomedicine, Life Sciences Institute, Guangxi Medical University, Nanning, China; ^3^ Department of Orthopaedics Trauma and Hand Surgery, The First Affiliated Hospital of Guangxi Medical University, Guangxi Medical University, Nanning, China; ^4^ Department of Orthopedics, The First People’s Hospital of Nanning, Nanning, China; ^5^ School of Biomedical Sciences, The University of Western Australia, Perth, WA, Australia; ^6^ Department of Pharmacy, People’s Hospital of Guangxi Zhuang Autonomous Region, Nanning, China; ^7^ Orthopaedics, Langdong Hospital of Guangxi Medical University, Guangxi Medical University, Nanning, China

**Keywords:** betulinic acid, osteoclast, receptor activator of nuclear factor‐κB ligand, osteoporosis, MAPK

## Abstract

Osteoclasts with elevated bone resorption are commonly present in postmenopausal osteoporosis, and other osteolytic pathologies. Therefore, suppressing osteoclast generation and function has been the main focus of osteoporosis treatment. Betulinic acid (BA) represents a triterpenoid mainly purified from the bark of Betulaceae. BA shows multiple biological activities, including antitumor and anti-HIV properties, but its effect on osteolytic conditions is unknown. Here, BA suppressed receptor activator of nuclear factor‐κB ligand (RANKL)‐associated osteoclastogenesis and bone resorptive function, as assessed by tartrate‐resistant acid phosphatase (TRAP) staining, fibrous actin ring generation, and hydroxyapatite resorption assays. Mechanistically, BA downregulated the expression of osteoclastic-specific genes. Western blot analysis revealed that BA significantly interrupted ERK, JNK and p38 MAPK activation as well as intracellular reactive oxygen species (ROS) production, thus altering c-Fos and NFATc1 activation. Corroborating the above findings in cell-based assays, BA prevented ovariectomy-associated bone loss in an animal model. In conclusion, these findings suggest that BA can inhibit osteoclast generation and function as well as the RANKL signaling pathway, and might be used for treating osteoclast-related osteoporosis.

## Introduction

Osteoporosis features decreased bone mass and strength, and characteristic bone microstructure degradation with increasing bone brittleness and fractures (Xie et al., 2019). It is commonly found among middle-aged and elderly individuals, with postmenopausal women accounting for the vast majority of cases; this disease represents a serious threat to human life and health (Lewiecki, 2011). Ovarian endocrine dysfunction in postmenopausal women, with estrogen level decline, leads to elevated bone resorption as well as reduced bone mass (Somjen et al., 2011; Feng et al., 2014). Increased differentiation of osteoclasts with elevated bone resorption is one of the causes of osteoporosis.

Osteoclasts are produced by myeloid progenitor cell differentiation of bone marrow mononuclear macrophages, with the formation of multinucleated giant cells (Adamopoulos & Mellins, 2015). Many signaling molecules contributing to osteoclast induction and proliferation play positive or negative regulatory roles; the most critical two factors include macrophage colony stimulating factor (M-CSF) and receptor activator of nuclear factor-κB ligand (RANKL), which contribute to osteoclast differentiation (Boyle et al., 2003; Boyce, 2013). M-CSF represents the most important key parameter promoting preosteoclast survival and enhancing the activity of the RANK and RANKL complex (Koehler et al., 2019). RANKL interacts with RANK on pre-osteoclasts, leading to TNF receptor-associated factor 6 (TRAF6) recruitment (Yang et al., 2019). Next, TRAF6 induces a series of downstream molecular networks such as the nuclear factor-κB (NF-κB) and mitogen-activated protein kinase (MAPK) pathways, involving extracellular signal-regulated kinase (ERK), c-Jun N-terminal kinases (JNK) and p38 mitogen-activated protein kinase (p38) (Yuan et al., 2015). These networks upregulate and activate transcription factors, including c-Fos and NFATc1, in a synergistic manner; meanwhile, NFATc1 constitutes the key molecule controlling the differentiation of osteoclasts (Takayanagi et al., 2002). Activating these downstream targets upregulates genes modulating OC differentiation and function such as those that encode tartrate-resistant acid phosphatase (TRAP), cathepsin K (CTSK) and matrix metalloproteinase 9 (MMP9), ultimately resulting in osteoclast maturation (Boyle et al., 2003). Additionally, reactive oxygen species (ROS) have been recognized as new messenger molecules for intracellular and extracellular regulation of osteoclast activity (Yip et al., 2005; Chen et al., 2019). RANKL induced signaling pathway is a therapeutic target for abnormal bone resorption.

**Figure 1:**
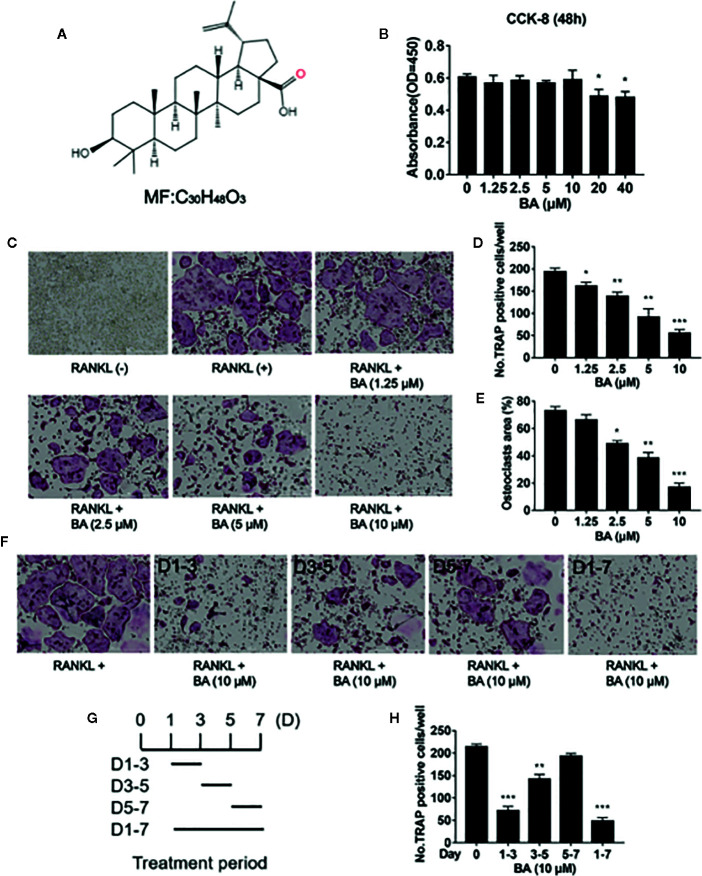
BA inhibits RANKL-associated osteoclastogenesis *in vitro*. **(A)** Structure of BA. **(B)** BMM viability as assessed by CCK-8. **(C)** Representative micrographs of TRAP stained samples. BMMs were cultured with M-CSF (50 ng/ml) and RANKL (100 ng/ml), and administered different BA amounts for 5 days. **(D, E)** Quantification of TRAP-positive osteoclasts (n = 3/group). Cells showing ≥3 nuclei were considered osteoclasts. BA dose‐dependently decreased RANKL-associated OC formation. **(F)** Osteoclastogenesis was promoted by RANKL (50 ng/ml), and cells were administered 10 μM BA for different times. **(G)** Treatment times of BA. **(H)** TRAP‐positive cells showing ≥3 nuclei were quantitated after BA administration. Data are mean ± SD. *p <0.05, **p <0.01, ***p <0.001 vs. RANKL-induced control group (no BA administration). Scale bar = 1000μM. BA, Betulinic acid; BMM, bone marrow macrophage; CCK-8, cell counting kit-8; RANKL, receptor activator of nuclear factor‐κB ligand; TRAP, tartrate-resistant acid phosphatase.

Natural products and derived molecules are indispensable in developing novel treatments for osteoporosis (Raut et al., 2019). Betulinic acid (BA) represents a natural pentacyclic triterpenoid (chemical formula shown in **Figure 1**), which widely exists in *Betula pubescens* and a variety of plants. BA has multiple biological effects, including anticancer, antidepressant, antimalarial, liver protective, anti-inflammatory, anti-HIV, anthelmintic, antibacterial and antifungal, and antioxidant activities (Saneja et al., 2018). Therefore, BA belongs to a class of potential drug lead compounds with important value for the development of new therapeutics. However, the role of BA in osteoporosis has been rarely studied. We hypothesized that BA could suppress osteoclasts and thus prevent osteoclast-associated osteoporosis.

Here, we tested BA’s effects on osteoclast generation and activation *in vitro* and explored the potential underlying mechanism. In addition, its therapeutic potential was assessed in a mouse model with osteolysis. Interestingly, BA could inhibit osteoclast generation and activity, decrease ROS production, downregulate c-Fos and NFATc1, and suppress the MAPK signaling pathway. Moreover, BA prevented ovariectomy (OVX)-induced osteoporosis *in vivo*. Taken together, the above findings provided novel insights into BA application for treating bone loss.

## Materials and Methods

### Materials

BA used in this experiment (≥98% purity) was provided by Chengdu Must Bio-Technology (Chengdu, China). α-Minimum essential medium (α‐MEM) and fetal bovine serum (FBS) were manufactured by Thermo Fisher Scientific (Thermo Fisher Scientific, Waltham, MA, USA). RANKL and M-CSF were provided by PeproTech (Princeton, NJ, USA). Cell Counting Kit-8 (CCK-8) assay and tartrate resistant acid phosphatase (TRAP) staining kits were provided by Sigma‐Aldrich (St. Louis, MO, USA). ROS detection kit was provided by Solarbio (Beijing, China). All primary and secondary antibodies were obtained from Cell Signaling (Danvers, MA, USA). DAPI and TRITC Phalloidin were obtained from Solarbio (Beijing, China).

### Cell Culture and Osteoclast Differentiation *In Vitro*

Stock solutions of BA (100 mM) in DMSO were kept at −20°C in darkness, and diluted with phosphate buffered solution (PBS) to prepare the corresponding concentration when it was used. To obtain mouse bone marrow macrophages (BMMs), bone marrow cavities of the femur and tibia of 6-week old female C57BL/6 mice were flushed. A total of eight mice were sacrificed in the *in vitro* cell experiment. The mice were purchased from the animal experiment center of Guangxi Medical University. All experimental procedures involving animals had approval from the Animal Care Committee of Guangxi Medical University. Upon filtration and centrifugation, the cell suspension was added into a T75 culture flask with α‐MEM supplemented by 10% FBS, 100 U/ml penicillin, 100 μg/ml streptomycin, and 50 ng/ml M-CSF (complete medium). About 3–4 days later, in order to induce osteoclast differentiation, BMMs underwent seeding into 96-well plates at 8 × 10^3^/well. After 24 h, the cells adhered to the plates, RANKL was added to the medium, in combination with various amounts of BA (1.25, 2.5, 5 and 10 µM) until mature osteoclasts were formed. Next, cells underwent fixation with 5% paraformaldehyde and staining using TRAP staining kit. Cells with >3 nuclei expressing TRAP were considered mature osteoclasts. BA’s effects on osteoclast differentiation at various stages were also determined. To this end, cells grown in 96-well plates in medium containing M-CSF (50 ng/ml) and RANKL (100 ng/ml), were administered BA on Days 1, 3, and 5, respectively, or continually on Days 1–7. Finally, osteoclasts in various wells were counted with ImageJ (NIH, Bethesda, MD, USA).

### Cell Proliferation and Cytotoxicity Assays

CCK-8 was performed for detecting the cytotoxic effects of BA. First, BMMs seeded in 96-well plates at 8 × 10^3^/well underwent incubation with M-CSF (50 ng/ml) for 14–16 h. This was followed by BA (1.25 to 40 µM) addition for stimulating the cells for 48 h. CCK-8 solution (10 µl/well) was added for incubation at 37°C in a humid environment with 5% CO _2_ for 2 h. Finally, absorbance was obtained at 450 nm on a microplate reader (Multiskan Spectrum; Thermo Lab Systems, USA).

### Intracellular ROS Generation Assay

ROS Assay Kit (Solarbio) was used to assess ROS levels. BMMs seeded in complete medium supplemented containing RANKL at 12 × 10^4^/well in 6-well plates, and incubated without or with BA (5 or 10 μM) for 3 days. Afterwards, the medium was replaced by dichloro-dihydro-fluorescein diacetate (DCFH-DA) in 1 ml FBS-free α-MEM (1/1,000). Incubation was carried out for 30 min (37°C, 5% CO_2)_ as directed by the manufacturer. DCFH-DA oxidation to DCF (intense fluorescence) by ROS was assessed under a fluorescence microscope. Data analysis was performed with ImageJ software (NIH).

### Hydroxyapatite Resorption Assay

To evaluate BA’s effects on osteoclast resorption, BMMs cultured in complete α-MEM were administered 50 ng/ml RANKL for 3 to 4 days till small osteoclasts were observed. Then, cells were digested and seeded into hydroxyapatite-coated Osteo Assay plate, followed by incubation with M-CSF and RANKL in combination or not with BA (5 and 10 μM) till observation of mature osteoclasts. Some wells per group underwent washing with 10% bleach for removing cells, and hydroxyapatite resorption areas were imaged under a Nikon microscope (Nikon, Japan). Other wells underwent fixation and staining for TRAP activity as described above for osteoclast numbering. The resorbed area/well and the percent of resorbed area/osteoclast were employed for quantitating osteoclast activity. Quantitation of resorption pit areas employed ImageJ (NIH, USA).

### Fibrous Actin (F‐Actin) Belt Formation Assay

BMMs were seeded onto a 96‐well plate at 8 × 10^3^/well and incubated in presence of M-CSF (50 ng/ml) for 14–16 h. To induce mature osteoclasts, M-CSF (as above) and RANKL (100 ng/ml) were added, in combination or not of different concentrations of BA (5 and 10 µM). Following 5 days of culture, osteoclasts underwent fixation (4% PFA; ambient conditions, 20 min) and permeabilization (0.25% Triton X-100, 5 min). Next, blocking was carried out by incubation with 3% bovine serum albumin (BSA) for 2 h. This was followed by incubation with Rhodamine-conjugated phalloidin (Thermo Fisher Scientific) for actin staining; DAPI was employed for counterstaining. Fluorescence images were acquired on an automated microscope named BioTek Inc. Instruments (Lionheart LX, USA).

### Quantitative Reverse‐Transcription Polymerase Chain Reaction (qRT-PCR)

The mRNA amounts of marker genes in osteoclasts were assessed by qRT-PCR. BMMs underwent seeding at 1 × 10^5^ in 6-well plates containing complete α-medium supplemented with M-CSF (50 ng/ml) and RANKL (100 ng/ml). Meanwhile, different BA levels (2.5, 5 and 10 μM) were added for 5 days. When osteoclast formation occurred in the positive group, total RNA extraction was carried out with TRIzol reagent (Life Technologies, Carlsbad, CA, USA). Revert Aid First Strand cDNA Synthesis Kit was employed for reverse transcription. qRT-PCR was carried out with SYBR Green PCR Master Mix, and GAPDH was employed for normalization. The comparative Ct (ΔCt) method was used for analysis. The primers tested are listed in **Table 1**.

**Table 1:** Primers for qRT-PCR.

**Gene**	**Primer sequence**		
**GAPDH**	Forward: Reverse:		5ʹ-AACTTTGGCATTGTGGAAGG-3ʹ; 5ʹ-ACACATTGGGGGTAGGAACA-3ʹ
**c-Fos**	Forward: Reverse:		5ʹ-GTGAAGACCGTGTCAGGAGG-3ʹ; 5ʹ-TCTGCGCAAAAGTCCTGTGT-3ʹ
**MMP9**	Forward: Reverse:		5ʹ-GAAGGCAAACCCTGTGTGTT -3ʹ; 5ʹ-AGAGTACTGCTTGCCCAGGA -3ʹ
**TRAcP**	Forward: Reverse:		5ʹ-ACGGCTACTTGCGGTTTCA-3ʹ 5ʹ-TCCTTGGGAGGCTGGTCTT-3ʹ
**CTSK**	Forward: Reverse:		5ʹ-AGGCGGCTATATGACCACTG-3ʹ 5ʹ-TCTTCAGGGCTTTCTCGTTC-3ʹ
**NFATc1**	Forward: Reverse:		5ʹ- GGTGCTGTCTGGCCATAACT-3ʹ 5ʹ- GAAACGCTGGTACTGGCTTC-3ʹ
**DC-STAMP**	Forward: Reverse:		5- TCTGCTGTATCGGCTCATCTC -3ʹ 5ʹ-ACTCCTTGGGTTCC TTGCTT-3ʹ

### Western Blot Analyses

BMM seeding was performed into 6-well plates at 5 × 10^5^/well, followed by a 14 to 16-h incubation. Then, the cells in FBS-free medium underwent a 3-hour culture, with or without BA pretreatment for 1 h. Radio-immunoprecipitation assay (RIPA) buffer was employed to extract total protein upon stimulation with RANKL (100 ng/ml) for 0, 5, 10, 20, 30, and 60 min respectively. For long-acting analyses of RANKL, BMMs (1 × 10^5^/well) underwent culture in complete medium with M-CSF (50 ng/ml) and RANKL (100 ng/ml), in combination or not with BA (10 µM) for 0, 1, 3, and 5 days, respectively. After cell lysis with chilled RIPA buffer (30 min), equal amounts of total protein in lysates were resolved by SDS-PAGE and electro-transferred onto nitrocellulose (NC) membranes. Blocking was carried out with 5% BSA, before incubation with specific primary antibodies for >12 h) at 4°C. Then, the membranes underwent incubation with corresponding secondary antibodies for 1 h at ambient. An Image Quant LAS-4000 Science Imaging System (GE Healthcare, USA) was employed to image immunoreactive bands, which were quantitated by the ImageJ software.

### Ovariectomy (OVX) Murine Model Establishment

We examined BA’s effects on bone loss in mice by constructing an osteoporosis model (OVX mice). All experimental procedures involving animals had approval from the Animal Care Committee of Guangxi Medical University. Female C57/BL6 mice (10 weeks old) were randomized into four groups (n = eight per group): sham, OVX + PBS, OVX + 5 mg/kg BA, and OVX + 10 mg/kg BA groups. Extraction of bilateral ovaries and partial fallopian tube resection were performed in all mice except the sham group; intraperitoneal injection of penicillin (once daily) was given 3 days after the operation for preventing wound infection. After 1 week, the OVX + 5 mg/kg BA and OVX + 10 mg/kg BA groups were intraperitoneally injected BA (5 and 10 mg/kg, respectively) for treatment every other day for six weeks. The mice were weighed before each treatment, the corresponding drug dose of mice were calculated according to the high and low concentration groups, diluted with PBS to make a volume of 0.5 ml liquid medicine injected into each mouse. The remaining two groups were given the same amount of PBS as vehicle controls. Upon treatment, euthanasia was performed by excessive anesthesia. The tibias were removed and submitted to fixation with 4% formalin, and prepared for micro-CT and histological analyses.

### Micro-CT and Histological Examinations

All collected mouse tibial specimens were scanned and analyzed by high‐resolution micro-CT (Sky scan; Bruker, USA). Quantitative morphometric assessments of bone features were carried out in a region of interest (ROI) delineated 0.5 mm below the growth plate. The bone indexes evaluated were: bone surface area/total volume (BS/TV), bone volume per tissue volume (BV/TV), trabecular thickness (Tb.Th), trabecular number (Tb.N) and trabecular separation (Tb.Sp). For histopathological analysis, tibia specimens after fixation underwent decalcification with 12% ethylenediaminetetraacetic acid (EDTA; pH 7.4) for 14 days, followed by paraffin embedding to assess TRAP activity or perform hematoxylin, eosin (H&E) staining and immunohistochemical staining. Sample imaging was carried out under a light microscope, and cells positive for TRAP were counted with ImageJ.

### Immunohistochemistry

The blank section of tibia tissues were dewaxed with xylene, then 3% H_2_O_2_ was used to block and inactivate endogenous peroxidase at 37°C for 15 min. After rinse with PBS, tissues sections were cooked in citric acid buffer for 10 min. The sections were incubated with primary antibody anti-RUNX2 (1:250; Abcam), anti-RANKL (1:200; Abcam) and anti-TNF-α (1:100; Abcam) at 4°C overnight, and then transferred to room temperature for 30 min. After washing with PBS, secondary antibody was added and incubated for 30 min at 37°C. 3’-Diaminobenzidine (DAB) reaction was performed, and hematoxylin was used as a counterstain. After photographing, Image-Pro Plus was used to measure the optical density of immunohistochemical positive expression in samples (Media Cybernetics, Inc., Rockville, MD).

### Statistical Analysis

Data with mean ± SD were assessed by Student’s t test (group pair comparisons) or one-way analysis of variance (ANOVA; multiple group comparisons). P <0.05 indicated statistical significance. SPSS (SPSS, USA) was also used for data analysis.

## Results

### BA Attenuates RANKL-Induced Osteoclast Formation

We first examined BA’s effects on cytotoxicity and cell survival by CCK-8. The results indicated that at 10 μM or less, BA had no BMM cytotoxicity after 48 h of treatment (**Figure 1B**). However, cytotoxicity was observed at concentrations of 20 μM and above. According to these results, the maximum concentration of 10 μM was selected for subsequent experiments. At non-cytotoxic doses, various BA amounts were supplemented to BMM cultures alongside RANKL induction to assess the effects on osteoclastogenesis. The results showed that BA dose-dependently decreased osteoclast quantities and size, and this effect was maximal at 10 µM, as shown in **Figure 1C**. Analyzing TRAP-positive osteoclasts with ≥3 nuclei, four BA concentrations (1.25, 2.5, 5, and 10 μM) showed significant differences in cell amounts and area ratio (**Figures 1D, E** **)**.

Next, BMMs were administered with BA (10 μM) for various times during stimulation by RANKL and M-CSF. **Figures 1F, G** depict the various times of BA administration and representative TRAP-staining images of osteoclasts. At Days 1 to 3, BA administration inhibited osteoclast differentiation to a greater extent in comparison with positive control-treated cells (**Figure 1F**). Mature osteoclasts (TRAP-positive and ≥3 nuclei) were quantitated (**Figure 1H**). In comparison with the early stage (Days 1 to 3), BA suppression of osteoclast differentiation was less pronounced compared with mid and late stages (Days 3 to 5 and 5 to 7, respectively). Jointly, the above findings demonstrated that BA dose-dependently reduced RANKL-associated osteoclast differentiation.

### BA Reduces Osteoclastic Hydroxyapatite Resorption and F-Actin Belt Generation Associated With RANKL

**Figure 2:**
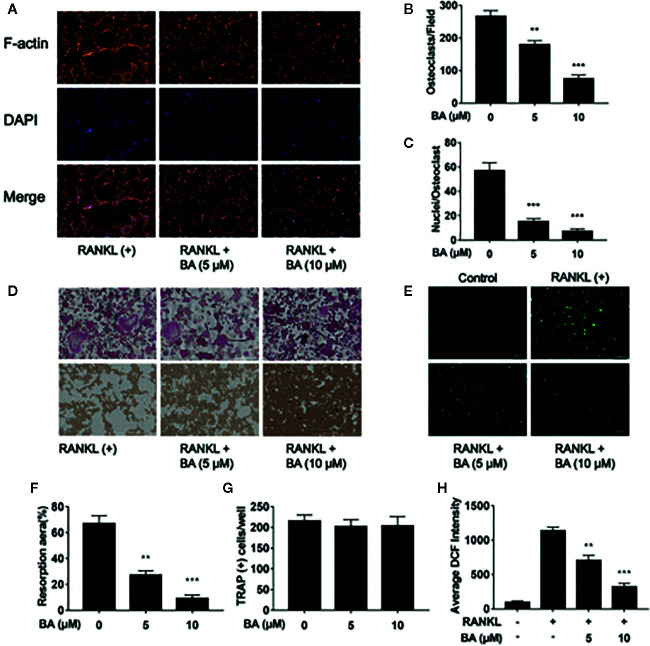
BA restrains F-actin belt formation and osteoclastic hydroxyapatite resorption triggered by RANKL while decreasing ROS levels in BMMs. **(A)** Representative images of formed F-actin belts as assessed by immunofluorescent staining with DAPI counterstaining. **(B)** Mean F-actin belt areas (n = 3). **(C)** Numbers of nuclei per osteoclast. **(D)** Representative micrographs of TRAP stained osteoclasts on hydroxyapatite coated plates. **(E)** Representative images for ROS production in presence or absence of BA in RANKL-treated BMMs. **(F, G)** Numbers of osteoclasts and areas of bone resorption assessed by the ImageJ software. **(H)** DCF intensities per cell indicating ROS levels. Data are mean ± SD. **p <0.01, ***p <0.001 vs. RANKL-induced control group (no BA administration). Scale bar = 1,000 μM, BA, Betulinic acid; DAPI, 4′,6‐diamidino‐2‐phenylindole; F-actin, fibrous actin.

Next, we assessed whether BA also impairs osteoclastic resorption. Mature osteoclasts were grown in hydroxyapatite-coated 96-well plates and administered BA (5 or 10 μM). In comparison with positive control-treated cells, hydroxyapatite resorption areas were reduced upon BA administration (**Figure 2D**). Based on TRAP staining, osteoclast amounts were observed in various wells (**Figure 2G**). Meanwhile, bone resorption areas were assessed with ImageJ. The results demonstrated that absorption areas after BA treatment were markedly decreased (**Figure 2F**). These findings suggested that BA decreased hydroxyapatite resorption areas by suppressing resorption function in osteoclasts instead of decreasing osteoclast amounts. Furthermore, BA’s effect was evaluated on F-actin belt generation, which is critically important in osteoclastic bone resorption. In this study, well-defined F-actin belts were found upon RANKL induction, while average cell nucleus numbers and F-actin belt areas were markedly decreased in BA treated groups (5 and 10 μM; **Figures 2A–C**). Notably, the areas of F-actin belts decreased by BA, indicating precursor cell fusion was suppressed.

### BA Attenuates ROS Production in BMMs

To detect ROS amounts in cells DCFH-DA was employed, and BA’s effect on RANKL-associated ROS production was determined. The results revealed that BA also decreased cell ROS levels in RANKL-associated osteoclast formation (**Figures 2E, F** **)**. In comparison with the RANKL-treatment only group, cells also administered BA showed remarkably reduced ROS-dependent conversion of DCFH-DA into DCF (**Figures 2E, F** **)**. Thus, these data indicated that BA effectively inhibited osteoclast differentiation and cellular ROS production after RANKL induction.

### BA Suppresses Osteoclastogenesis via RANKL-Activated MAPK Signaling

**Figure 3:**
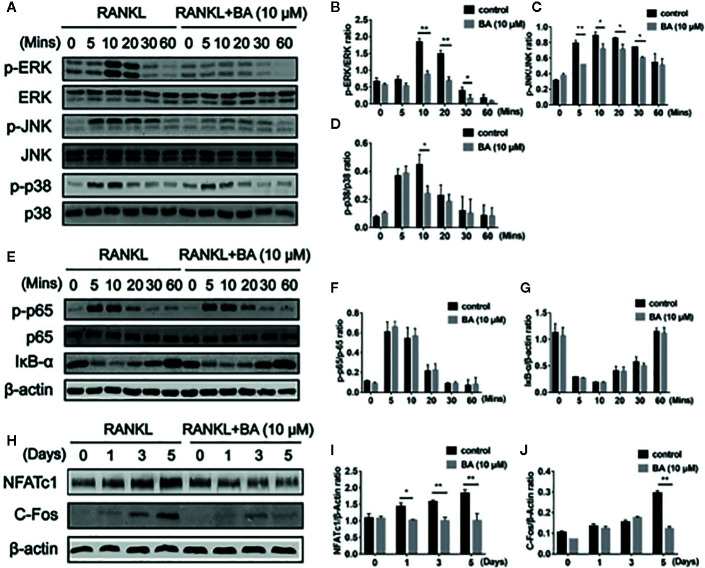
BA suppresses osteoclastogenesis through RANKL-induced signaling pathways. **(A)** Representative immunoblot images for BA’s effects on ERK, JNK and p38 phosphorylation after RANKL induction. BMMs were administered with RANKL for various times in combination or not with 10 μM BA. **(B–D)** Ratios of phosphorylated ERK, JNK and p38 to total ERK, JNK and p38, respectively (n = 3). **(E)** Immunoblot images for BA’s effects on p65 degradation and IκB-α phosphorylation associated with RANKL at various time points. **(F, G)** Ratios of phosphorylated p65 and IκB-α to total p65 and β-actin, respectively. (n = 3 per group). **(H)** Immunoblot images for BA’s effects on NFATc1 and c-Fos protein levels. **(I, J)** Ratios of NFATc1 and c-Fos to β-actin (n = 3/group). Data are mean ± SD. *p <0.05, **p <0.01 vs. RANKL-induced control group (no BA administration). BA, Betulinic acid.

**Figure 4:**
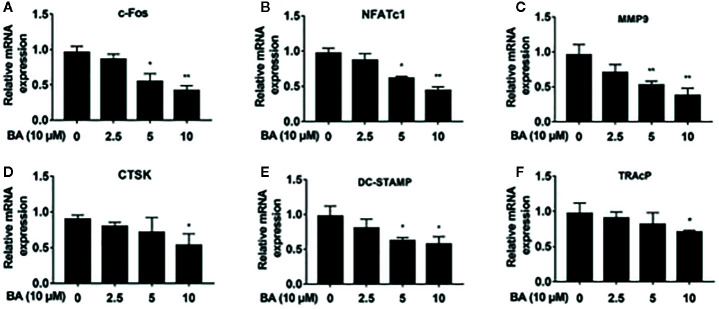
BA downregulates the expression of osteoclast-specific genes. **(A–F)** The qRT‐PCR assay detected mRNA amounts of osteoclastogenesis-associated marker genes, including c-Fos, NFATc1, MMP9, CTSK, DC-STAMP and TRAP. (n = 3/group). Data are mean ± SD. *p <0.05, **p <0.01 vs. RANKL-induced control group (no BA administration). BA, Betulinic acid; PCR, polymerase chain reaction.

To further investigate the molecular mechanism of BA on RAKNL-induced osteoclast generation, osteoclast-associated pathways induced by RANKL were explored. Immunoblot demonstrated that the ratio of phosphorylated ERK to total ERK was markedly decreased after treatment with BA at 10, 20 and 30 min, respectively (**Figures 3A, B** **)**. In addition, the ratio of phosphorylated p38 to total p38 in osteoclasts was altered just at 10 min (**Figures 3A, D** **)**. Meanwhile, phosphorylated JNK to total JNK ratio was overtly reduced at 5, 10, 20 and 30 min, (**Figures 3A, C** **)**. BA’s effect on ERK p38 phosphorylation was unclear (**Figures 4A, B, D** **)**. Concerning the NF-κB pathway, a non-significant upregulation of IκBα and anti-phosphorylation of p65 were found (**Figures 3E–G**). The above findings suggested BA suppressed MAPK signaling, specifically inhibiting ERK, JNK and p38 activities, but not altering NF-κB signaling.

### BA Abrogates RANKL‐Associated NFATc1 and c‐Fos Activation, and Downregulates NFATc1’s Target Genes

BA’s effects were assessed on the activation of NFATc1 and c-Fos, as downstream effectors of MAPK signaling. After BA administration, NFATc1 and c-Fos protein amounts were markedly decreased upon RANKL stimulation for 3 days (**Figures 3H–J**). These findings suggested that BA could downregulate multiple osteoclast marker genes. As shown by qRT-PCR, NFATc1’s target genes, such as c-Fos, NFATc1, MMP9, CTSK, DC-STAMP and TRAcP, were dose-dependently downregulated by BA at the mRNA level (**Figures 4A–F**). The above findings indicated that BA could reduce NFATc1 and c-Fos protein amounts, downregulating important osteoclast-specific genes controlled by NFATc1.

### BA Administration Protects Against Bone Loss in Ovariectomy (OVX) Mouse Models

**Figure 5:**
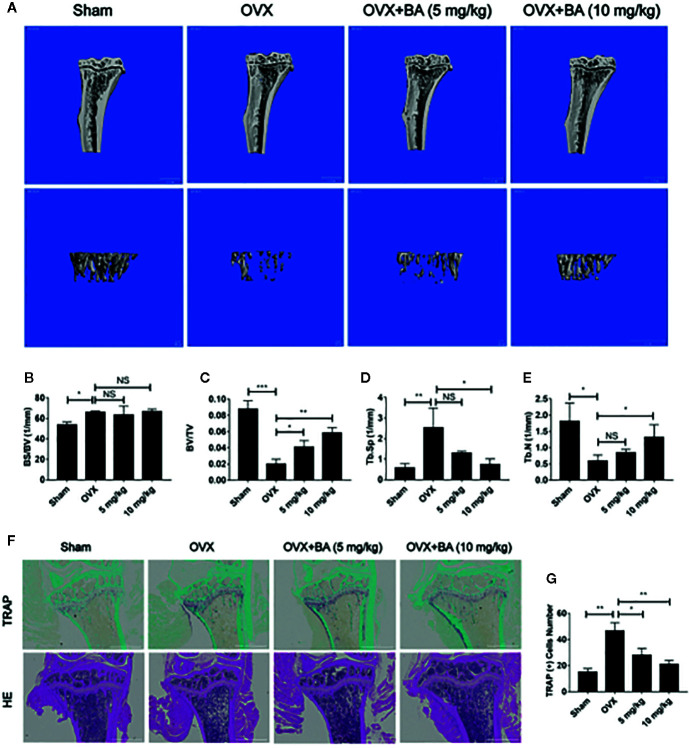
BA prevents bone loss in mice with ovariectomized (OVX)-induced osteoporosis. **(A)** Representative 3D reconstruction micro-CT images of tibial micro-architecture. **(B–E)** Quantitative micro-CT assessment of trabecular bone volume fraction (BV/TV), bone surface area/total volume (BS/TV), specific bone surface (BS/BV) and trabecular number (Tb.N). **(F)** Representative photographs of tibia samples after TRAP and H&E staining. **(G)** Quantitation of TRAP‐positive cells (n = 3). Data are mean ± SD. *p <0.05, **p <0.01, ***p <0.001 vs. OVX group. BA, Betulinic acid; OVX, ovariectomized.

**Figure 6:**
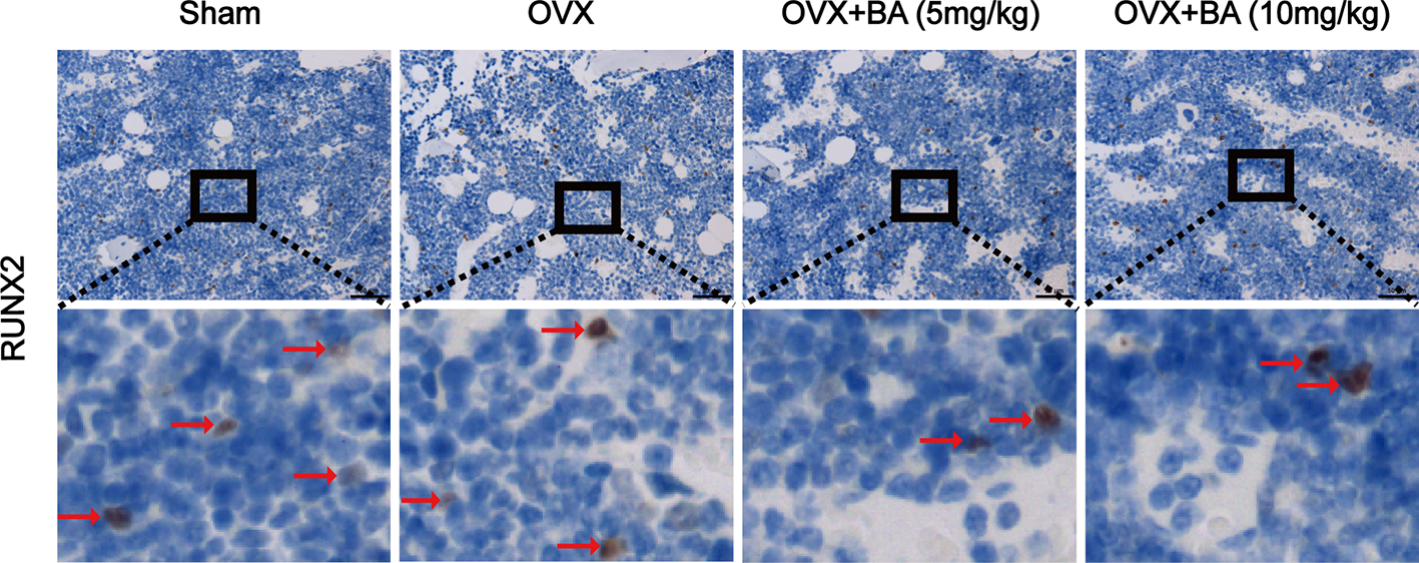
BA does not affect ovariectomized (OVX)-induced expression of RUNX2 *ex vivo*. The expression of RUNX2 protein in tibial tissues in each group was detected by immunohistochemistry, and the expression of RUNX2 in the images were brown or russet and indicated by the red arrow. RUNX2, runt-related transcription factor 2.

Based on the above effects of BA in cultured cells, we then assessed BA’s impact on bone loss in mice. OVX-induced bone loss represents a common model of estrogen deficiency-associated osteoporosis, which is broadly employed for mimicking human postmenopausal osteoporosis. In this study, compared with sham animals, OVX mice showed tibial bones with overt bone loss, substantial decrease of bone volume (BV/TV) and deteriorated trabecular bone architecture i.e., reduced Tb.Th and Tb.N (**Figures 5A–E**). Administration of BA, particularly at high levels (10 mg/kg) remarkably protected the animals from OVX-associated bone loss and trabecular deterioration. This beneficial effect of BA in OVX-associated bone loss was verified histopathologically. TRAP sections showed elevated bone volume and improved trabecular bone architecture as a result of reduced osteoclast activity on the bone surface (Oc.S/BS) following BA treatment (**Figures 5F, G** **)**. H&E staining revealed that bone volume and surface in OVX mice were both well-preserved after BA treatment in comparison with the non-BA group (**Figures 5F, G** **)**. The results of immunohistochemical assay showed that BA had no significant effect on the expression of RUNX2 in tibial tissues (**Figure 6**). The expression of RANKL and TNF-α in tibial tissue was also assessed by immunohistochemistry, and BA was found to attenuate the expression RANKL and TNF-α (**Supplementary Figure 1**), consistently with its inhibitory effect on osteoclastogenesis. Collectively, these findings revealed BA as a potent molecule for treating OVX-induced bone loss, *via* reduction of both osteoclast amounts and bone resorption.

## Discussion

Osteoporosis is a systemic osteopathy featuring reduced bone mass accompanied by elevated risk of fracture (Fuggle et al., 2019). Most commonly found in the elderly and postmenopausal women, postmenopausal osteoporosis (PMOP) accounts for the majority of cases. In postmenopausal women, there is a sudden drop in estrogen levels, causing osteoclast bone resorption to surpass osteoblast bone formation (Feng et al., 2014; Xin et al., 2018). Current FDA-approved treatment interventions mainly include two categories: (1) inhibitors of bone absorption, e.g. calcitonin bisphosphonates, and estrogen selective estrogen receptor modulators and (2) inducers of bone formation, including parathyroid hormone, growth hormones and statins. However, their side effects and long-term efficacy are concerning (Khosla & Hofbauer, 2017). Therefore, there is an urgent need for new and safer drugs for preventing or treating bone loss associated diseases. The current understanding of natural products indicates that they constitute a possible source of potential bone protective agents. Multiple biological and pharmacological *in vitro* and experimental studies have revealed that a variety of natural products could maintain or promote bone health with potentially beneficial effects and reduced side effects (Chaugule et al., 2019). BA from birch (*B. pubescens*) and a variety of plants, as a natural pentacyclic triterpene compound, has anti-human immunodeficiency virus (HIV), antibacterial, anti-inflammatory, antimalarial and anti-herpes simplex virus 1 (HSV-1) properties (Amiri et al., 2019). However, whether and how BA affects osteoclasts is unknown.

Osteoporosis represents a systemic skeletal pathology featuring reduced bone mass and bone tissue alteration at the microstructural level, with increased bone brittleness (Compston et al., 2019). This involves reduced bone mass with excessive activation of osteoclasts (e.g., Paget’s disease) commonly seen in osteoporosis and bone metastases (e.g., multiple myeloma) (Tsukasaki and Takayanagi, 2019). In cases with postmenopausal osteoporosis, bone mass loss is mainly caused by reduced estrogen accompanied by increased osteoclasts and elevated bone resorptive activity (Kou et al., 2019). In this study, we demonstrated that BA in mice with osteoporosis induced by OVX prevented bone loss. In addition, multiple assays were performed to determine BA’s effects on osteoclast differentiation *in vitro* and bone resorption associated with RANKL induction. We found that BA did not significantly reduced the number of osteoclasts cultured in hydroxyapatite coated plates. These results indicated that BA by inhibiting hydroxyapatite absorption reduces osteoclast function. In addition, BA markedly reduced the formation of F-actin belts that are required for osteoclast bone resorption. At the molecular level, BA downregulated CTSK and MMP-9 that contribute to osteoclast F-actin belt generation as well as bone resorption (Dodds et al., 2001; Wilson et al., 2009; Tanaka et al., 2013). Furthermore, qRT-PCR found downregulation of genes involved in osteoclast differentiation, including c-Fos, NFATc1, MMP-9, CTSK, DC-STAMP and TRAP.

The transcription factor NF-κB is critical for multiple cellular pathways; therefore, its suppression is considered an efficient approach for inhibiting osteoclast formation and bone resorption (Xu et al., 2009). As a result, multiple researchers have targeted NF-κB for osteopathy. Differentiation of osteoclasts from hematopoietic progenitors requires M-CSF and RANKL, which induce the proliferation progenitors/precursors and differentiation effects on more mature cells (Anesi et al., 2019). After RANKL stimulation of osteoclast progenitor cells and osteoclast surface RANK, intracellular segments of RANK recruit TRAF6 (Park et al., 2017), an essential upstream effector of the RANKL signal transduction pathway, which further mediates intracellular signal cascade after activation, including promoting the phosphorylation and subsequent degradation of NF-κB by inducing IKK phosphorylation, releasing NF-κB, initiating the transcription of corresponding genes, and ultimately regulating osteoclast differentiation and bone absorption (Boyce et al., 2015). IκBα degradation and nuclear translocation of p65 represent critical steps of NF-κB signaling induction in the generation of osteoclasts (Xu et al., 2009). Here, we observed no marked suppressive effects of BA on IκBα degradation or p65 phosphorylation, indicating the anti-osteoclastic effects of BA are not caused by NF-κB inactivation. Therefore, we hypothesized that another pathway, MAPK signaling, which encompasses JNK, p38, and ERK, is also induced by phosphorylation and in combination with NF-κB modulates the transcription of genes that initiate osteoclast formation (Choi et al., 2017). In pre-osteoclasts, ERK (ERK 1 and 2) and JNK (JNK1, 2 and 3) isoforms mostly control cell proliferation and apoptosis, respectively (Boyle et al., 2003; Ikeda et al., 2008; Lee et al., 2016). Of all p38 isoforms (α, β, γ, and δ), p38α shows high amounts in pre-osteoclasts as well as mature osteoclasts, with critical functions in osteoclast differentiation and bone resorption (Bohm et al., 2009). ERK signaling essentially comprises three protein kinases. Raf activation through interaction with Raf induces the MAPKKs MEK1 and MEK2, which control ERK1 and ERK2 activation (Kinoshita et al., 1997; Raman et al., 2007), resulting in the phosphorylation of multiple downstream effectors, e.g., c-Fos and NFATc1. Meanwhile, c-Fos and NFATc1, as essential transcription factors in osteoclastogenesis, are regulated by GM-CSF-induced ERK signaling (Lee et al., 2009). The osteoclastogenic protein RANKL induces the JNK pathway *via* TRAF6, promoting differentiation in osteoclasts (Ikeda et al., 2008; Otero et al., 2008). RANKL-induced activated JNK regulates the phosphorylation of the transcription factor c-Jun, which binds to c-Fos to form a complex that constitutes a critical transcription factor in the generation of osteoclasts (Grigoriadis et al., 1994; Ikeda et al., 2008). In addition, JNK signaling upregulates calcium/calmodulin-dependent protein kinase (CaMK), c-Fos, and NFATc1, which contribute to osteoclast lineage commitment (Chang et al., 2008; Park et al., 2015). The p38 pathway is important in the modulation of osteoclast formation and maturation, and consequently controls bone resorption and remodeling (Boyle et al., 2003; Cong et al., 2017). RANKL binding to RANK results in p38 phosphorylation in pre-osteoclasts *via* TRAF6, promoting osteoclast differentiation (Matsumoto et al., 2000; Boyle et al., 2003). Meanwhile, p38 activated *via* the RANKL-TAK1-MKK6 pathway phosphorylates NF-κB p65 on Serine 536, upregulating NF-κB and NFATc1 (Huang et al., 2006). As shown above, BA overtly reduced ERK phosphorylation and also inhibited p38 and JNK. Thus, MAPK signaling is important in BA-related inhibition of osteoclast differentiation. The above finding indicates that BA may suppress or modulate the activity of an upstream inducer of the ERK, p38, and JNK pathway.

NFATc1 and c-Fos, two critical downstream effectors of MAPK signaling, upregulate genes related to osteoclast differentiation, including TRAP (Acp5), MMP-9, DC-STAMP, CTSK, and so on (Lee et al., 2018). As shown above, both NFATc1 and c-Fos were downregulated by BA as shown by Western blot, confirming that BA downregulates proteins contributing to osteoclast differentiation. The elevation of intracellular ROS content affects osteoclast differentiation and the activation of MAPKs, including ERK, p38 and JNK, in multiple cell lines (Wang et al., 2016; Ohyama et al., 2018; Chen et al., 2019). Our biochemical assays indeed demonstrated that BA inhibited cellular ROS production during RANKL-associated osteoclastogenesis. Thus, the observed decrease of NFATc1 amounts after BA treatment may result from a combination of inhibited ERK, JNK and p38 MAPK signaling suppression and decreased intracellular ROS levels. Whether BA directly affects MAPK signaling or inhibits ROS production, indirectly affecting MAPK signaling, deserves further investigation. In addition, the mechanisms by which BA inhibits ROS production should be determined in future studies.

As the key functional cells of bone formation, osteoblasts are responsible for the synthesis, secretion and mineralization of bone matrix, it is very important for the maintenance of normal bone mass. Runx2 is an important regulatory factor in the differentiation of osteoblasts. Numerous *in vitro* and *in vivo* studies have found that overexpression of Runx2 can induce differentiation of mesenchymal stem cells into osteoblasts (Franceschi et al., 2007). Therefore, the expression of RUNX2 protein in tibial tissue was detected by immunohistochemical staining, and the results showed that BA had no effect on the expression of RUNX2, which indirectly indicated that BA had no effect on osteoblasts. We also detected the expression of RANKL and TNF-α in tibial tissue by immunohistochemistry, and found that BA attenuated their expression, in line with its suppressing effect on osteoclast formation and bone resorption.

In conclusion, BA exerts overt suppressive effects on osteoclast differentiation *in vitro* mainly through MAPK and NFATc1 signaling pathways, preventing bone loss in the mouse OVX model. Developing BA derivatives could provide novel and better candidate drugs for treating osteoporosis and other bone diseases.

## Data Availability Statement

All datasets generated for this study are included in the article/**Supplementary Material**.

## Ethics Statement

The animal study was reviewed and approved by The Animal Care & Welfare Committee of Guangxi Medical University.

## Author Contributions

YY and JZ were responsible for the guidance and examination of the project. JW and YLi were responsible for cell and animal experiments. JW was responsible for data analysis and article writing. QL, YLa, CW, KT, LW, and CL helped to search the literature and do animal experiments. JX helped to revise the article.

## Funding

This project is supported in part by Co-innovation Centre for Bio-Medicine, Innovation Team of Tissue Repair and Reconstruction. This project was also supported by the National Natural Science Foundation of China (NSFC No. 81501910, 81702186), the Natural Science Foundation of Guangxi Province (2015GXNSDA139019, 2017GXNSFBA198061).

## Conflict of Interest

The authors declare that the research was conducted in the absence of any commercial or financial relationships that could be construed as a potential conflict of interest.

## References

[B1] Adamopoulos I. E. Mellins E. D. (2015). Alternative pathways of osteoclastogenesis in inflammatory arthritis. Nat. Rev. Rheumatol. 11 (3), 189–194. 10.1038/nrrheum.2014.198 25422000 PMC4346500

[B2] Amiri S. Dastghaib S. Ahmadi M. Mehrbod P. Khadem F. Behrouj H. (2019). Betulin and its derivatives as novel compounds with different pharmacological effects. Biotechnol. Adv. 38 (2020), 10.1016/j.biotechadv.2019.06.008 31220568

[B3] Anesi A. Generali L. Sandoni L. Pozzi S. Grande A. (2019). From Osteoclast Differentiation to Osteonecrosis of the Jaw: Molecular and Clinical Insights. Int. J. Mol. Sci. 20 (19). 10.3390/ijms20194925 PMC6801843 31590328

[B4] Bohm C. Hayer S. Kilian A. Zaiss M. M. Finger S. Hess A. (2009). The alpha-isoform of p38 MAPK specifically regulates arthritic bone loss. J. Immunol. 183 (9), 5938–5947. 10.4049/jimmunol.0901026 19828631

[B5] Boyce B. F. Xiu Y. Li J. Xing L. Yao Z. (2015). NF-kappaB-Mediated Regulation of Osteoclastogenesis. Endocrinol. Metab. (Seoul) 30 (1), 35–44. 10.3803/EnM.2015.30.1.35 25827455 PMC4384681

[B6] Boyce B. F. (2013). Advances in osteoclast biology reveal potential new drug targets and new roles for osteoclasts. J. Bone Miner. Res. 28 (4), 711–722. 10.1002/jbmr.1885 23436579 PMC3613781

[B7] Boyle W. J. Simonet W. S. Lacey D. L. (2003). Osteoclast differentiation and activation. Nature 423 (6937), 337–342. 10.1038/nature01658 12748652

[B8] Chang E. J. Ha J. Huang H. Kim H. J. Woo J. H. Lee Y. (2008). The JNK-dependent CaMK pathway restrains the reversion of committed cells during osteoclast differentiation. J. Cell Sci. 121 (Pt 15), 2555–2564. 10.1242/jcs.028217 18650497

[B9] Chaugule S. Kashipathi Sureshbabu S. Dakave S. Krishna C. M. Chaudhari P. Indap M. (2019). Hexane Fraction of Turbo brunneus Inhibits Intermediates of RANK-RANKL Signaling Pathway and Prevent Ovariectomy Induced Bone Loss. Front. Endocrinol. (Lausanne) 10, 608. 10.3389/fendo.2019.00608 PMC6742724 31555218

[B10] Chen K. Qiu P. Yuan Y. Zheng L. He J. Wang C. (2019). Pseurotin A Inhibits Osteoclastogenesis and Prevents Ovariectomized-Induced Bone Loss by Suppressing Reactive Oxygen Species. Theranostics 9 (6), 1634–1650. 10.7150/thno.30206 31037128 PMC6485188

[B11] Choi J. H. Han Y. Kim Y. A. Jin S. W. Lee G. H. Jeong H. M. (2017). Platycodin D Inhibits Osteoclastogenesis by Repressing the NFATc1 and MAPK Signaling Pathway. J. Cell Biochem. 118 (4), 860–868. 10.1002/jcb.25763 27739107

[B12] Compston J. E. McClung M. R. Leslie W. D. (2019). Osteoporosis. Lancet 393 (10169), 364–376. 10.1016/s0140-6736(18)32112-3 30696576

[B13] Cong Q. Jia H. Li P. Qiu S. Yeh J. Wang Y. (2017). p38alpha MAPK regulates proliferation and differentiation of osteoclast progenitors and bone remodeling in an aging-dependent manner. Sci. Rep. 7, 45964. 10.1038/srep45964 28382965 PMC5382695

[B14] Dodds R. A. James I. E. Rieman D. Ahern R. Hwang S. M. Connor J. R. (2001). Human osteoclast cathepsin K is processed intracellularly prior to attachment and bone resorption. J. Bone Miner. Res. 16 (3), 478–486. 10.1359/jbmr.2001.16.3.478 11277265

[B15] Feng J. Liu S. Ma S. Zhao J. Zhang W. Qi W. (2014). Protective effects of resveratrol on postmenopausal osteoporosis: regulation of SIRT1-NF-kappaB signaling pathway. Acta Biochim. Biophys. Sin. (Shanghai) 46 (12), 1024–1033. 10.1093/abbs/gmu103 25377437

[B16] Franceschi R. T. Ge C. Xiao G. Roca H. Jiang D. (2007). Transcriptional regulation of osteoblasts. Ann. N Y Acad. Sci. 1116, 196–207. 10.1196/annals.1402.081 18083928

[B17] Fuggle N. R. Curtis E. M. Ward K. A. Harvey N. C. Dennison E. M. Cooper C. (2019). Fracture prediction, imaging and screening in osteoporosis. Nat. Rev. Endocrinol. 15 (9), 535–547. 10.1038/s41574-019-0220-8 31189982

[B18] Grigoriadis A. E. Wang Z. Q. Cecchini M. G. Hofstetter W. Felix R. Fleisch H. A. (1994). c-Fos: a key regulator of osteoclast-macrophage lineage determination and bone remodeling. Science 266 (5184), 443–448. 10.1126/science.7939685 7939685

[B19] Huang H. Ryu J. Ha J. Chang E. J. Kim H. J. Kim H. M. (2006). Osteoclast differentiation requires TAK1 and MKK6 for NFATc1 induction and NF-kappaB transactivation by RANKL. Cell Death Differ. 13 (11), 1879–1891. 10.1038/sj.cdd.4401882 16498455

[B20] Ikeda F. Matsubara T. Tsurukai T. Hata K. Nishimura R. Yoneda T. (2008). JNK/c-Jun signaling mediates an anti-apoptotic effect of RANKL in osteoclasts. J. Bone Miner. Res. 23 (6), 907–914. 10.1359/jbmr.080211 18251700

[B21] Khosla S. Hofbauer L. C. (2017). Osteoporosis treatment: recent developments and ongoing challenges. Lancet Diabetes Endocrinol. 5 (11), 898–907. 10.1016/s2213-8587(17)30188-2 28689769 PMC5798872

[B22] Kinoshita T. Shirouzu M. Kamiya A. Hashimoto K. Yokoyama S. Miyajima A. (1997). Raf/MAPK and rapamycin-sensitive pathways mediate the anti-apoptotic function of p21Ras in IL-3-dependent hematopoietic cells. Oncogene 15 (6), 619–627. 10.1038/sj.onc.1201234 9264402

[B23] Koehler M. II Hartmann E. S. Schluessel S. Beck F. Redeker J. II Schmitt B. (2019). Impact of Periprosthetic Fibroblast-Like Cells on Osteoclastogenesis in Co-Culture with Peripheral Blood Mononuclear Cells Varies Depending on Culture System. Int. J. Mol. Sci. 20 (10). 10.3390/ijms20102583 PMC6567687 31130703

[B24] Kou J. Zheng X. Guo J. Liu Y. Liu X. (2019). MicroRNA-218-5p relieves postmenopausal osteoporosis through promoting the osteoblast differentiation of bone marrow mesenchymal stem cells. J. Cell Biochem. 121 (2). 10.1002/jcb.29355 31478244

[B25] Lee M. S. Kim H. S. Yeon J. T. Choi S. W. Chun C. H. Kwak H. B. (2009). GM-CSF regulates fusion of mononuclear osteoclasts into bone-resorbing osteoclasts by activating the Ras/ERK pathway. J. Immunol. 183 (5), 3390–3399. 10.4049/jimmunol.0804314 19641137

[B26] Lee K. Chung Y. H. Ahn H. Kim H. Rho J. Jeong D. (2016). Selective Regulation of MAPK Signaling Mediates RANKL-dependent Osteoclast Differentiation. Int. J. Biol. Sci. 12 (2), 235–245. 10.7150/ijbs.13814 26884720 PMC4737679

[B27] Lee K. Seo I. Choi M. H. Jeong D. (2018). Roles of Mitogen-Activated Protein Kinases in Osteoclast Biology. Int. J. Mol. Sci. 19 (10). 10.3390/ijms19103004 PMC6213329 30275408

[B28] Lewiecki E. M. (2011). New targets for intervention in the treatment of postmenopausal osteoporosis. Nat. Rev. Rheumatol. 7 (11), 631–638. 10.1038/nrrheum.2011.130 21931340

[B29] Matsumoto M. Sudo T. Saito T. Osada H. Tsujimoto M. (2000). Involvement of p38 mitogen-activated protein kinase signaling pathway in osteoclastogenesis mediated by receptor activator of NF-kappa B ligand (RANKL). J. Biol. Chem. 275 (40), 31155–31161. 10.1074/jbc.M001229200 10859303

[B30] Ohyama Y. Ito J. Kitano V. J. Shimada J. Hakeda Y. (2018). The polymethoxy flavonoid sudachitin suppresses inflammatory bone destruction by directly inhibiting osteoclastogenesis due to reduced ROS production and MAPK activation in osteoclast precursors. PloS One 13 (1), e0191192. 10.1371/journal.pone.0191192 29342179 PMC5771597

[B31] Otero J. E. Dai S. Foglia D. Alhawagri M. Vacher J. Pasparakis M. (2008). Defective osteoclastogenesis by IKKbeta-null precursors is a result of receptor activator of NF-kappaB ligand (RANKL)-induced JNK-dependent apoptosis and impaired differentiation. J. Biol. Chem. 283 (36), 24546–24553. 10.1074/jbc.M800434200 18567579 PMC2528995

[B32] Park H. Noh A. L. Kang J. H. Sim J. S. Lee D. S. Yim M. (2015). Peroxiredoxin II negatively regulates lipopolysaccharide-induced osteoclast formation and bone loss via JNK and STAT3. Antioxid. Redox Signal 22 (1), 63–77. 10.1089/ars.2013.5748 25074339 PMC4270137

[B33] Park J. H. Lee N. K. Lee S. Y. (2017). Current Understanding of RANK Signaling in Osteoclast Differentiation and Maturation. Mol. Cells 40 (10), 706–713. 10.14348/molcells.2017.0225 29047262 PMC5682248

[B34] Raman M. Chen W. Cobb M. H. (2007). Differential regulation and properties of MAPKs. Oncogene 26 (22), 3100–3112. 10.1038/sj.onc.1210392 17496909

[B35] Raut N. Wicks S. M. Lawal T. O. Mahady G. B. (2019). Epigenetic regulation of bone remodeling by natural compounds. Pharmacol. Res. 147, 104350. 10.1016/j.phrs.2019.104350 31315065 PMC6733678

[B36] Saneja A. Arora D. Kumar R. Dubey R. D. Panda A. K. Gupta P. N. (2018). Therapeutic applications of betulinic acid nanoformulations. Ann. N Y Acad. Sci. 1421 (1), 5–18. 10.1111/nyas.13570 29377164

[B37] Somjen D. Katzburg S. Sharon O. Grafi-Cohen M. Knoll E. Stern N. (2011). The effects of estrogen receptors alpha- and beta-specific agonists and antagonists on cell proliferation and energy metabolism in human bone cell line. J. Cell Biochem. 112 (2), 625–632. 10.1002/jcb.22959 21268084

[B38] Takayanagi H. Kim S. Koga T. Nishina H. Isshiki M. Yoshida H. (2002). Induction and activation of the transcription factor NFATc1 (NFAT2) integrate RANKL signaling in terminal differentiation of osteoclasts. Dev. Cell 3 (6), 889–901. 10.1016/s1534-5807(02)00369-6 12479813

[B39] Tanaka H. Tanabe N. Kawato T. Nakai K. Kariya T. Matsumoto S. (2013). Nicotine affects bone resorption and suppresses the expression of cathepsin K, MMP-9 and vacuolar-type H(+)-ATPase d2 and actin organization in osteoclasts. PloS One 8 (3), e59402. 10.1371/journal.pone.0059402 23555029 PMC3598738

[B40] Tsukasaki M. Takayanagi H. (2019). Osteoimmunology: evolving concepts in bone-immune interactions in health and disease. Nat. Rev. Immunol. 19 (10), 626–642. 10.1038/s41577-019-0178-8 31186549

[B41] Wang H. Li D. Hu Z. Zhao S. Zheng Z. Li W. (2016). Protective Effects of Green Tea Polyphenol Against Renal Injury Through ROS-Mediated JNK-MAPK Pathway in Lead Exposed Rats. Mol. Cells 39 (6), 508–513. 10.14348/molcells.2016.2170 27239812 PMC4916403

[B42] Wilson S. R. Peters C. Saftig P. Bromme D. (2009). Cathepsin K activity-dependent regulation of osteoclast actin ring formation and bone resorption. J. Biol. Chem. 284 (4), 2584–2592. 10.1074/jbc.M805280200 19028686 PMC2629117

[B43] Xie Y. Zhang L. Xiong Q. Gao Y. Ge W. Tang P. (2019). Bench-to-bedside strategies for osteoporotic fracture: From osteoimmunology to mechanosensation. Bone Res. 7, 25. 10.1038/s41413-019-0066-7 31646015 PMC6804735

[B44] Xin Z. Jin C. Chao L. Zheng Z. Liehu C. Panpan P. (2018). A Matrine Derivative M54 Suppresses Osteoclastogenesis and Prevents Ovariectomy-Induced Bone Loss by Targeting Ribosomal Protein S5. Front. Pharmacol. 9, 22. 10.3389/fphar.2018.00022 29441015 PMC5797611

[B45] Xu J. Wu H. F. Ang E. S. Yip K. Woloszyn M. Zheng M. H. (2009). NF-kappaB modulators in osteolytic bone diseases. Cytokine Growth Factor Rev. 20 (1), 7–17. 10.1016/j.cytogfr.2008.11.007 19046922

[B46] Yang L. Zhang B. Liu J. Dong Y. Li Y. Li N. (2019). Protective Effect of Acteoside on Ovariectomy-Induced Bone Loss in Mice. Int. J. Mol. Sci. 20 (12). 10.3390/ijms20122974 PMC6627387 31216684

[B47] Yip K. H. Zheng M. H. Steer J. H. Giardina T. M. Han R. Lo S. Z. (2005). Thapsigargin modulates osteoclastogenesis through the regulation of RANKL-induced signaling pathways and reactive oxygen species production. J. Bone Miner. Res. 20 (8), 1462–1471. 10.1359/JBMR.050324 16007343

[B48] Yuan F. L. Xu R. S. Jiang D. L. He X. L. Su Q. Jin C. (2015). Leonurine hydrochloride inhibits osteoclastogenesis and prevents osteoporosis associated with estrogen deficiency by inhibiting the NF-kappaB and PI3K/Akt signaling pathways. Bone 75, 128–137. 10.1016/j.bone.2015.02.017 25708053

