# Supplementary material for: Betulinic Acid Protects From Bone Loss in Ovariectomized Mice and Suppresses RANKL-Associated Osteoclastogenesis by Inhibiting the MAPK and NFATc1 Pathways
Source: Front Pharmacol. 2020 Jul 7;11:1025. doi: 10.3389/fphar.2020.01025 (PMC7358641; doi:10.3389/fphar.2020.01025)
Supplement: Supplementary file 1 [file Image_1.pdf]

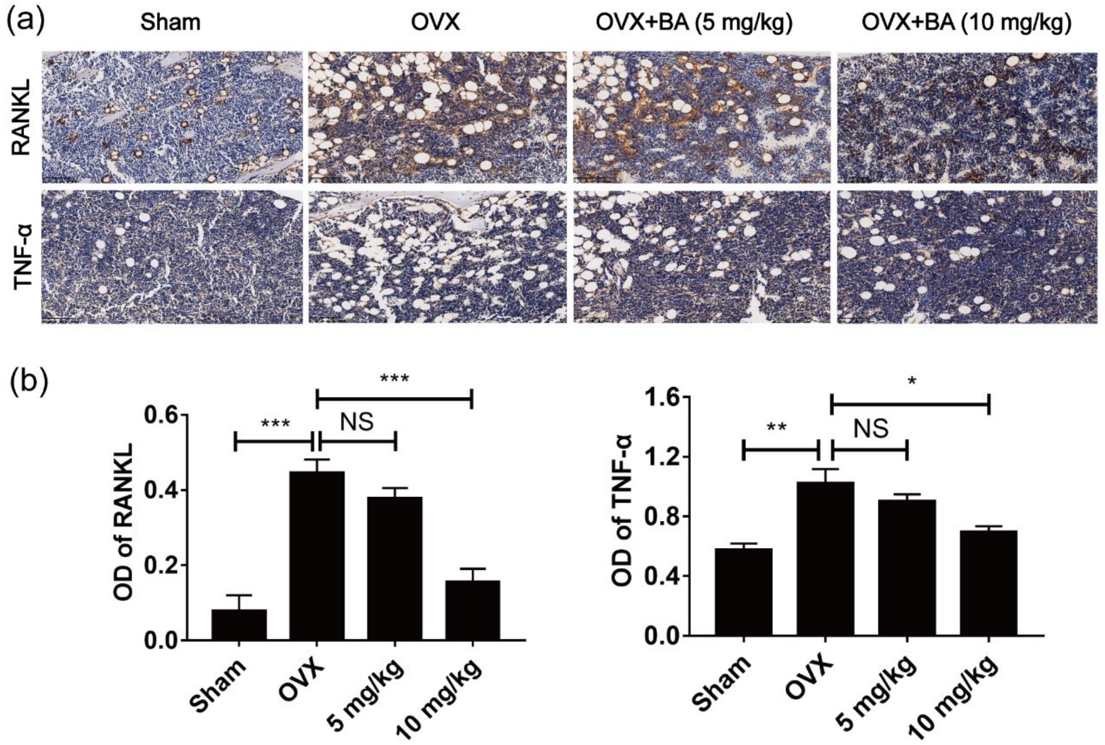

Supplementary Figure 1. BA suppresses ovariectomized (OVX)-induced expression of RANKL and TNF-α ex vivo.

(a) The expression of RANKL protein and TNF-α protein in tibial tissues in each group was detected by immunohistochemistry, and the expression of RANKL and TNF-α in the images were brown or russet. (b) Image Pro-Plus 6.0 was used to measure the optical density of immunohistochemical positive expression in samples (n=3). Data are mean±SD. *$p<0.05$, **$p<0.01$, ***$p<0.001$ vs. OVX group. RANKL, receptor activator of nuclear factor-κB ligand; TNF-α, tumor necrosis factor alpha.
